# Correction to “Chromatin Topology Reconfiguration Orchestrates Thermotolerant Male Fertility via GhAL5 in Cotton”

**DOI:** 10.1002/advs.75669

**Published:** 2026-05-11

Yanlong Li^1^, Jing Yang^2^, Weiran Wang^2^, Chunyang Zuo^1^, Liuling Pei^1^, Yizan Ma^1^, Rui Zhang^1^, Yaru Fan^1^, Huanhuan Ma^1^, Yawei Li^1^, Ruizhen Liu^1^, Shuangxia Jin^1^, Longfu Zhu^1^, Jie Kong^2^, Xianlong Zhang^1^, Ling Min^1^, “Chromatin Topology Reconfiguration Orchestrates Thermotolerant Male Fertility via GhAL5 in Cotton,” *Advanced Science* (Weinheim, Baden‐Wurttemberg, Germany), 13 no. 14 (2026), e07766. https://doi.org/10.1002/advs.202507766

**Figure 2D**:

In the published version, the sample label “H05” was inadvertently duplicated and displayed as “84021” in the legend. The second set of samples should have been labeled as “H05.” The published version of Figure 2D and the corrected version are provided below. The specific labeling error in the published figure is indicated with red arrows for clarity.


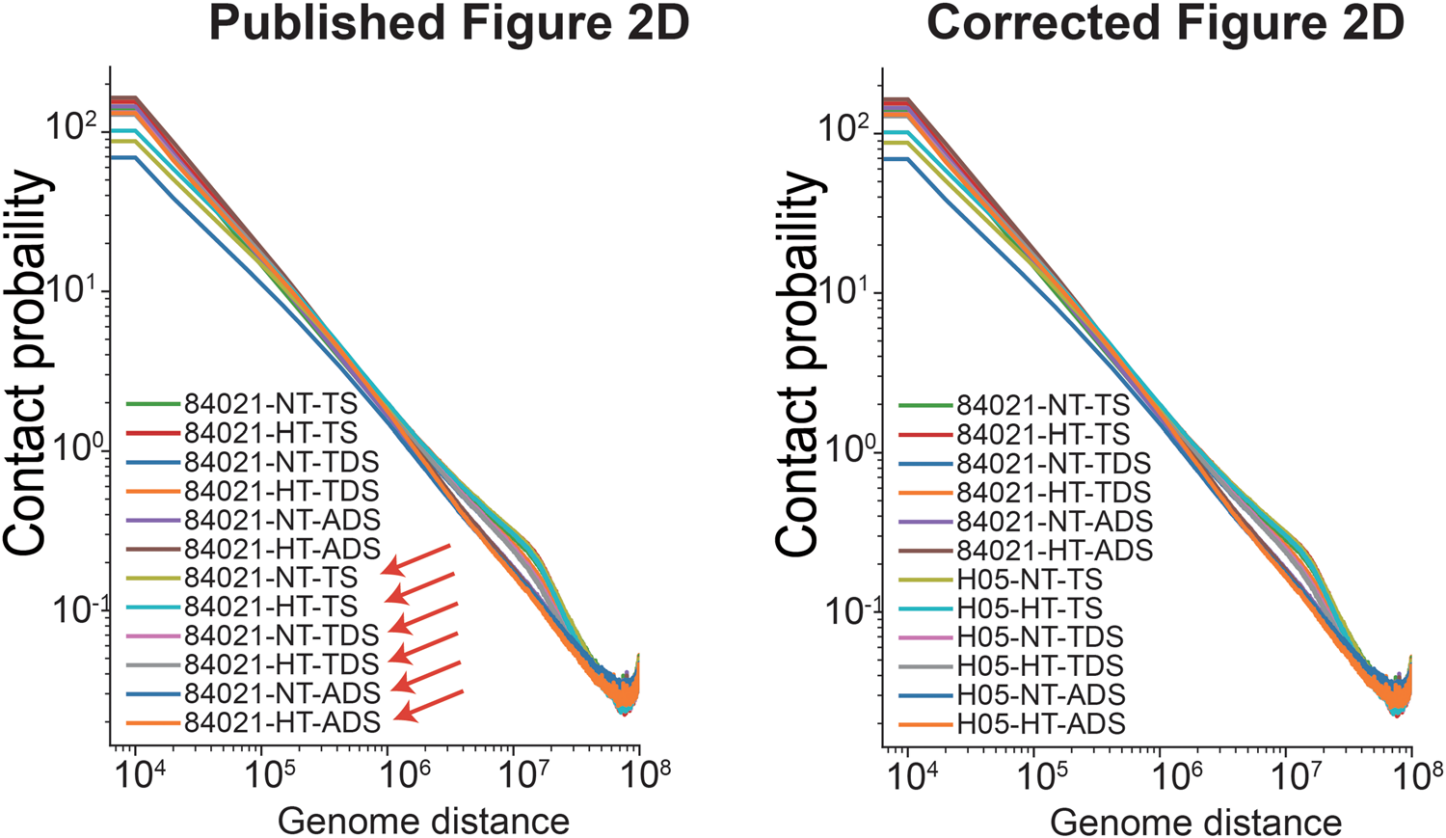


**Figure 3B**:

In the published version, the treatment label for H05 under heat treatment (HT) was incorrectly displayed as “NT.” The correct label should be “HT.” The published version of Figures 3B and the corrected version are provided below. The specific labeling error in the published figure is indicated with red arrows for clarity.


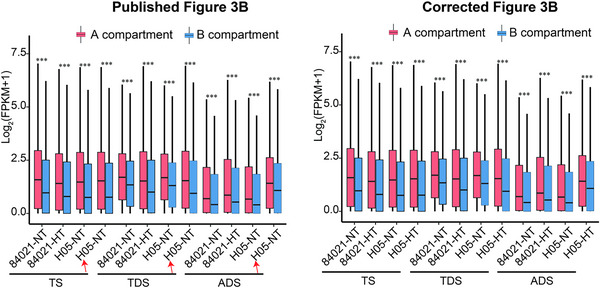


**Figure 6F**:

In the published version, samples under HT conditions were incorrectly labeled as “NT” for both 84021 and H05. The labels have now been corrected to “HT.” The published version of Figures 6F and the corrected version are provided below. The specific labeling error in the published figure is indicated with red arrows for clarity.


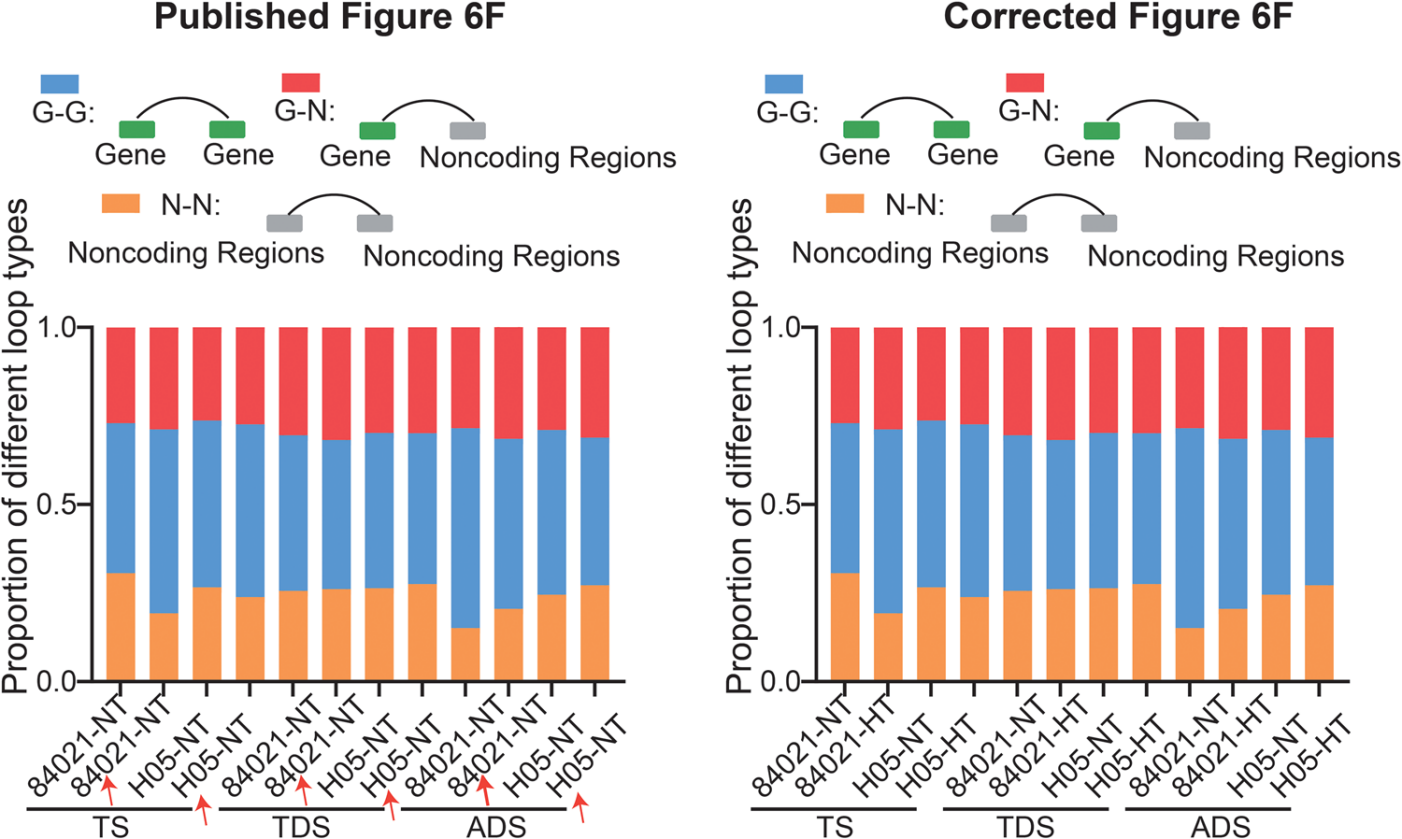


**Supplementary Figure S13B**:

For H05 samples, the HT treatment groups were incorrectly labeled as “NT” in both left and right panels. The treatment labels have been corrected. The published version of Supplementary Figure S13B and the corrected version are provided below. The specific labeling error in the published figure is indicated with red arrows for clarity.


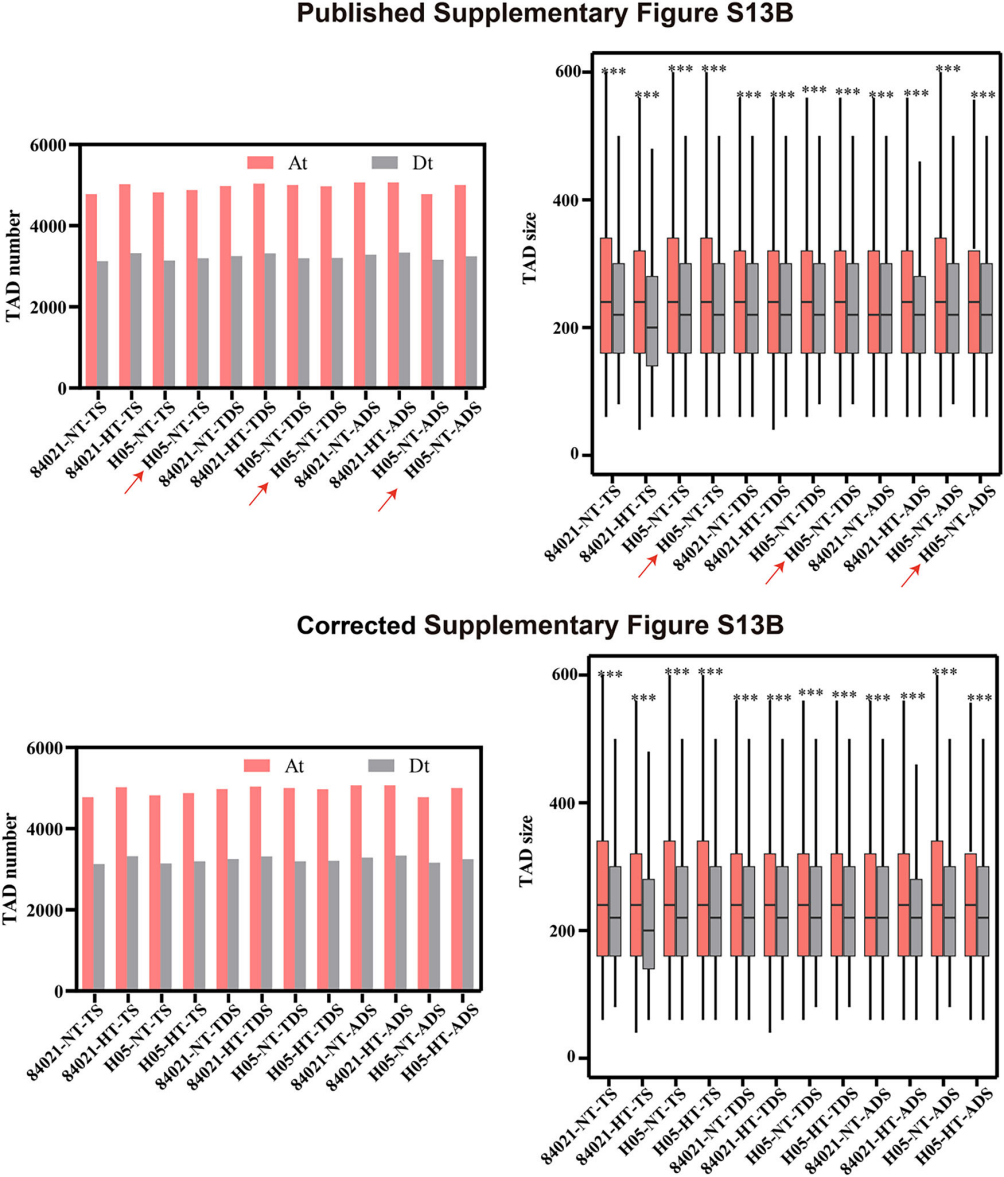


**Supplementary Figure S17**:

For H05 samples, the HT treatment groups were incorrectly labeled as “NT” in the figure panels. The treatment labels have been corrected. The published version of Supplementary Figure S17 and the corrected version are provided below. The specific labeling error in the published figure is indicated with red arrows for clarity.


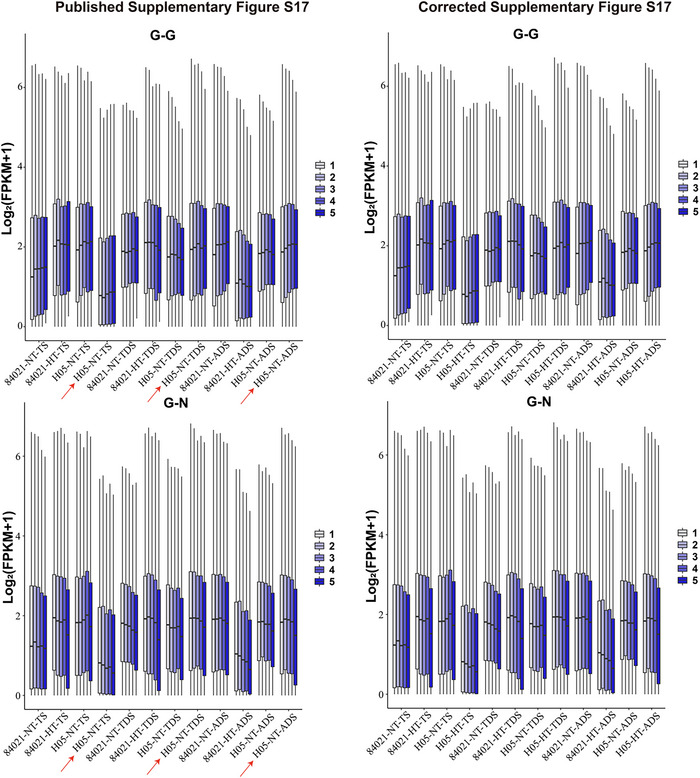


These corrections do not affect the interpretation of the data or the conclusions of the study.

We apologize for this error.

